# Loss of *G*2 subunit of vacuolar-type proton transporting ATPase leads to *G*1 subunit upregulation in the brain

**DOI:** 10.1038/srep14027

**Published:** 2015-09-10

**Authors:** Nobuyuki Kawamura, Ge-Hong Sun-Wada, Yoh Wada

**Affiliations:** 1 Faculty of Pharmaceutical Sciences, Doshisha Women’s College of Liberal Arts, Kohdo, Kyotanabe, Kyoto 610-0395, Japan; 2 Division of Biological Sciences, Institute of Scientific and Industrial Research, Osaka University, Mihogaoka 8-1, Ibaraki, Osaka 567-0047, Japan

## Abstract

Vacuolar-type ATPase (V-ATPase) is a primary proton pump with versatile functions in various tissues. In nerve cells, V-ATPase is required for accumulation of neurotransmitters into secretory vesicles and subsequent release at the synapse. Neurons express a specific isoform (*G*2) of the *G* subunit of V-ATPase constituting the catalytic sector of the enzyme complex. Using gene targeting, we generated a mouse lacking functional *G*2 (*G*2 null), which showed no apparent disorders in architecture and behavior. In the *G*2-null mouse brain, a *G*1 subunit isoform, which is ubiquitously expressed in neuronal and non-neuronal tissues, accumulated more abundantly than in wild-type animals. This *G*1 upregulation was not accompanied by an increase in mRNA. These results indicate that loss of function of neuron-specific *G*2 isoform was compensated by an increase in levels of the *G*1 isoform without apparent upregulation of the *G*1 mRNA.

Vacuolar-type ATPase (V-ATPase) is a proton translocating pump driven by chemical energy released by ATP hydrolysis. It is present in the internal membranes of all eukaryotic cells, where it translocates protons from the cytosol into organelle lumen, resulting in an acidic environment within^1^. Various cellular functions of vital importance, including transport of solutes, membrane trafficking, and hydrolysis of macromolecules in digestive compartments are highly dependent on the acidic conditions and chemiosmotic potential energy created by V-ATPase^2^. Accordingly, loss of its activity results in embryonic lethality at the peri-implantation stage of early embryogenesis^3^. V-ATPase is also implicated in highly differentiated functions in various cells and tissues^4^,^5^,^6^. For example, V-ATPase-dependent acidification in the endocytic pathway is essential for defense against pathogens by macrophages as well as T-cell mediated antigen processing^7^,^8^. V-ATPase also contributes to establish the acidic environment outside the differentiated cells such as bone resorbing osteoclasts, epithelial cells in the kidney, epididymis, and other tissues^5^,^9^,^10^,^11^,^12^.

V-ATPase is a multi-subunit complex that comprises 2 major functional sectors, V1 and V0^13^. V1 is involved in ATP hydrolysis and consists of 8 subunits (*A*, *B*, *C*, *D*, *E*, *F*, *G,* and *H*). V0 serves as a proton pathway across the membranes and consists of 5 subunits (*a*, *c*, *c*’, *d*, and *e*). The catalytic sites, composed of the *A* and *B* subunits are connected to the V0 proton-transporting sector by a central stalk composed of the *D* subunit and 3 elongated structures, made up of the subunits *E* and *G*^14^. These stalks are involved in the energy coupling of V1 and V0, which undergo association/dissociation cycles by which V-ATPase activity changes in response to physiological conditions^15^. The stalk structures play an important role in the reversible assembly/disassembly of the enzyme complex^16^.

The *G* subunit in the mouse is a small 13-kDa protein, composed of 118 amino acids. *G* subunit interacts with the *E* subunit to form a rod-like structure connecting the V1 catalytic and V0 transporting domains. Therefore, *G* is thought to be important for regulation of V-ATPase activities^14^,^17^. The mammalian genome contains 3 distinctive genes for this subunit, namely *ATP6V1G1*, *ATP6V1G2*, and *ATP6V1G3*^18^. *G*1 isoform, encoded by *ATP6V1G1* is expressed in various tissues and cells, while, *G*2 and *G*3 isoforms show restricted tissue-specific distribution in the brain and kidney, respectively^18^,^19^. The neurons express the unique *G*2 isoform in addition to the ubiquitously distributed *G*1 isoform, whereas other cells including astrocytes and glia only express the ubiquitous *G*1 isoform^19^.

V-ATPase is involved in multiple neuronal functions. Abundant in synaptic vesicles, it provides chemiosmotic energy for loading neurotransmitters^20^,^21^,^22^. Additionally, V-ATPase participates in membrane fusion between the synaptic vesicle and presynaptic plasma membrane^23^. Tissue-specific expression of the *G*2 isoform raise an intriguing argument that it may have a dedicated function in the neural system. However, the physiological significance of this unique expression pattern of the *G*2 isoform has not been elucidated to date. No genetic impairment of the *ATP6V1G2* locus in human or any animal model has been identified to date; therefore, phenotypic consequences of the loss of neuron-specific *G*2 remains to be experimentally determined. In this study, we introduced a null mutation into the mouse *Atp6v1g2* locus. The loss of the *G*2 function did not result in any developmental defect or obvious behavioral abnormality until adulthood. We found that deletion of *G*2 function induced the up-regulation of *G*1 subunit levels in the brain, suggesting that the amounts of V-type ATPase subunits are strictly regulated. This up-regulation was not accompanied by an increase in levels of the *Atg6v1g1* transcripts, implying the existence of a post-transcriptional regulatory mechanism.

## Results

**Figure 1:**
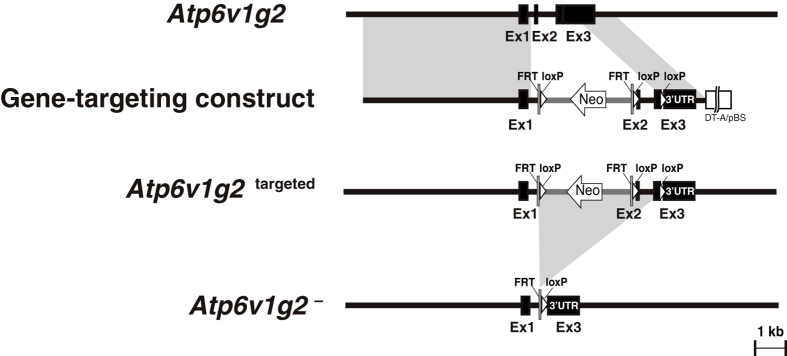
Modification of the mouse *Atp6v1g2* locus encoding V-ATPase *G*2 subunit. The embryonic stem cell line R1 was transfected with a gene-targeting construct carrying a *lox* P-*Frt*-*npt*-*lox* P-*Frt* cassette and the third *lox* P, in intron 1–2 and the 3′-UTR of *Atp6v1g2*, respectively. Chimeric animals were generated by injecting the homologous recombinants into the blastocoels of C57Bl/6 embryos. Mice carrying the *Atp6v1g2*^targeted^ allele were crossed with the FvB/N-Tg(EIIa-cre)C5379Lmgd/J strain, expressing *Cre* recombinase during early development to remove exon 2 (Ex2) and a part of exon 3 (Ex3), to generate *Atp6v1g2*^+/−^ mice. *Atp6v1g2*^+/−^ mice were further crossed with wild-type C57Bl/6 mice to remove the *Cre* transgene and a cohort of *Atp6v1g2*^+/−^ mice was established. 3′-UTR; 3′-untranslated region, Neo; neomycin resistance cassette.

The mammalian genome contains 3 isoforms of the V-ATPase *G* subunit, the catalytic sector of the proton pump complex. Transcription of the *Atp6v1g2* locus is highly restricted to the neurons. In order to elucidate the physiological relevance of this *G* subunit isoform, we created a genetically modified mouse. We generate a modified version of *Atp6v1g2*, in which *lox* P-*Frt*-*npt*-*lox* P-*Frt* was placed in intron 1–2, and the third *lox* P in the 3′-untranslated region (Fig. 1). Cre-mediated recombination between the remotest *lox* P sequences produced an allele that was missing the exons 2 and 3, resulting in a truncated coding sequence comprising only residues 1 through 27; therefore, it was highly likely to be a null allele (Fig. 1), which we designated as *Atp6v1g2*^−^.

Mice heterozygous for the null allele (*Atp6v1g2*^+/−^) were normal and fertile. They were intercrossed to generate *Atp6v1g2*^−^/^−^ progeny, which appeared at the expected ratio upon the genotyping at 3 weeks after birth, indicating that the loss of the *G*2 isoform did not result in embryonic or post-natal lethality (Table 1). They did not show any discernible behavioral differences among littermates with mixed genetic background of 129Sv (from ES cells), C57Bl6 (breeding spouse), and FvB (Cre-expressing strain). We were unable to distinguish *G*2-null mice from heterozygous or wild-type littermates in a cohort maintained for more than a year, unless genotypes were determined by PCR.

**Figure 2:**
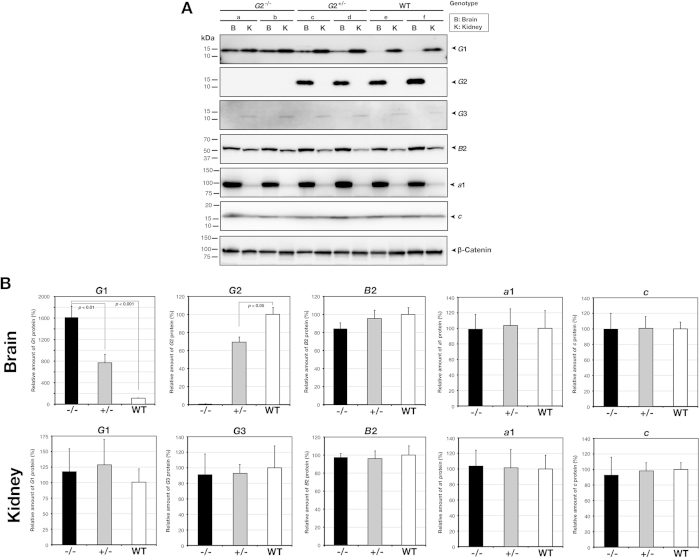
Immunoblot analysis of *G* subunit isoforms in the brain and kidney. (**A**) lysates (10 μg/lane) prepared from the brain and kidneys of *Atp6v1g2*^*−*/*−*^ (lanes a,b) mice, *Atp6v1g2*^+/−^ (lanes c,d) and wild-type (lanes e,f) mice, and then they were subjected to gel electrophoresis in the presence of sodium dodecyl sulfate. *G*1, *G*2, *G*3 isoforms, *a*1, *c* and *B*2 subunit were probed by the specific antibodies. V-ATPase *a*1, *c*, and *B*2 subunits represent the quantity of total V-ATPase in tissues. β-catenin was also detected for standardization of loadings. (**B**) the band density was quantified using a luminoimager and relative quantity of subunit isoforms was determined. The levels in the wild-type tissues were defined as 100%. The bars represent standard deviations of 3 independent experiments using 4 samples of each genotype.

We previously reported a set of polyclonal antibodies, each specifically recognizing *G*1, *G*2, and *G*3 isoforms^19^,^24^. Using isoform-specific antibodies, we examined the expression of *G*1, *G*2, and *G*3 in the brain and kidney of wild-type and *G*2-null mice by immunoblot (Fig. 2A). The *G*1 isoform was present in both, the brain and kidneys of the wild-type mouse, but *G*2 was detected only in the brain and not the kidneys (Fig. 2A). As previously reported^18^, *G*3 isoform was specifically expressed in the kidney (Fig. 2A). In the mice homologous for the null allele (*Atp6v1g2*^*−*/*−*^ mice), no *G*2 was detected in the brain lysate (Fig. 2A,B), thus underscoring that the *Atp6v1g2*^−^ allele was indeed null. The quantity of V-ATPase *B*2 subunit, the component of the V1 sectors was similar in the wild-type and mutant brains. The amounts of subunits *a*1 and *c*, the components of the V0 membrane intrinsic sector, were accumulated similarly in the brains of all the genotypes (Fig. 2). These results implied that the total quantity of V-ATPase accumulated remained unchanged. The *G*1 isoform in the mutant brain appeared more abundant as compared to that in the wild-type brain (Fig. 2). In contrast, the quantity of *G*1 isoform in the kidney, where no *G*2 isoform is normally produced, was similar in the wild-type and mutants (Fig. 2). This observation indicated that an increase of *G*1 subunit occurred specifically in the brain, where the *G*2 subunit was highly expressed. The compensatory increase of *G*1 associated with the genetic loss of *G*2 isoform suggested that the absence of *G*2 subunit leads to an increase in *G*1 subunit in the brain. In contrast to the compensation between *G*1 and *G*2 isoforms, *G*3 remained absent in the mutant brain, showing that loss of *G*2 did not alter the expression specificity of the kidney-specific isoform.

**Figure 3:**
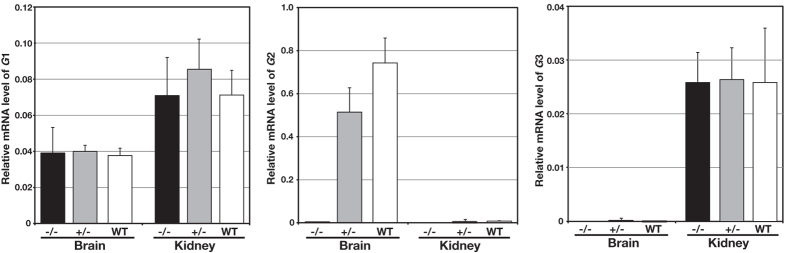
Expression of *G* subunit isoforms in the brain and kidneys. The expression levels of G-subunit isoform transcripts were determined by quantitative PCR. Total RNA was obtained from the brain and kidney, reverse-transcribed, and then amplified in the presence of primer pairs, specific for each isoform. Amplification was monitored by SYBR green fluorescence using a Prism 7000 detector. Relative expression levels were determined after normalization to *Gapdh* transcripts. The expression levels were determined in 4, 3, and 4 mice of wild-type, *Atp6v1g2*^+/−^, and *Atp6v1g2*^*−*/*−*^, respectively. The bars represent standard deviations.

We investigated whether the observed increase in the quantity of *G*1 isoform reflected an up-regulation at the transcription level by examining the levels of *Atp6v1g1, Atp6v1g2*, and *Atp6v1g3* transcripts. Total RNA was isolated from the brain and kidney of the wild-type, heterozygous (*Atp6v1g2*^+/−^), and homozygous (*Atp6v1g2*^*−*/*−*^) mutant animals, cDNAs were reverse-transcribed, and quantitative real-time PCR analysis was performed. mRNA quantity was estimated by the efficiency calibrated model^25^ using *Gapdh* transcripts as standard (Fig. 3). In the wild-type animals, *G*1 and *G*2 transcripts were detected at high levels in the brain, whereas *G*2 transcripts were found in the brain but only a minor fraction in the kidney. *G*3 transcripts were strictly restricted to the kidney, while this expression pattern and levels in the kidney were unchanged across all genotypes. In the heterozygous and homozygous mutant brains, the levels of *G*1 transcripts remained unchanged. The fold changes of *G*1 expression in heterozygous (*Atp6v1g2*^+/−^), and homozygous (*Atp6v1g2*^*−*/*−*^) mutant brains when compared with wild-type were 1.27 and 1.02, respectively, indicating that the transcription and stability of mRNA play a minor role, if any, for upregulation of *G*1 subunit isoform. The quantity of *G*2 transcripts showed a moderate decrease in the heterozygous mice (0.65-folds of that in the wild-type), indicating that transcripts levels were proportional to the gene dosage.

## Discussion

The variety of functions performed by V-ATPase in different cellular contexts are attributed to the structural heterogeneity of the enzyme complex, comprising of multiple subunit isoforms. The higher eukaryotes, both, vertebrates and invertebrates develop such diversity by having evolved different subunit isoforms in both, V1 and V0 sectors^26^. This suggests that diverse structural variation is required for more elaborate physiological demands in multicellular organisms.

In mammals, there are 3 distinctive isoforms of the *G* subunit. In a previous study, we showed that V-ATPase complexes immunoisolated by anti-*G*1 and anti-*G*2 antibodies exhibit similar kinetic profiles for ATP hydrolysis^19^. However, the ubiquitously expressed *G*1 isoform can complement the loss of Vma10, a yeast orthologue of the *G* subunit, whereas the brain-specific isoform *G*2 does not rescue Vma10 deficiency in yeast. This difference shown in a heterologous expression system suggests that *G*1 and *G*2 interact differently with other subunits of yeast V-ATPase^19^.

The *G* subunit forms a rod-shaped structure with the *E* subunit. This rod constitutes a stator, connecting the membrane-peripheral V1 and membrane-intrinsic V0 sectors^14^. V-ATPase undergoes reversible dissociation/association of the V1 and V0 sectors, through which, ATP hydrolysis and proton translocation can be regulated in response to physiological conditions^27^. The stator structures play a central role in the reversible disassembly of the V0V1-ATPase complex. Physiological relevance of this dissociation/association is established in yeast and invertebrate organisms, however, its role in mammals remains to be explored. Structural signatures of *G*, *E*, and *C* subunits which have 3, 2, and 3 isoforms, respectively, might confer differential characteristics of assemble/disassemble cycles in mammalian enzymes. This, genetically modified animals with specific loss of the *G*2 subunit could provide a model to explore this possibility.

We found that the quantity of V-ATPase seemed to be maintained in spite of loss of *G*2 subunit isoforms, suggesting the presence of a mechanism regulating the total activity and/or quantity of V-ATPase in the cell. This argument has been repeatedly raised by previous observations with regards to specific impairment of *a*- and *B*-subunit isoforms. *B*1-deficient renal epithelium can be rescued by increasing activity of V-ATPase *B*2 isoform^28^. This compensation is achieved by recruiting *B*2-containing V-ATPase from intracellular membranes to the cell surface, not necessarily involving transcriptional and/or translational up regulation^29^. Acidification of phagosomes requires the *a*3 isoform. The lumen of phagosomes in *a*3-deficient mice still exhibits bafilomycin-sensitive, V-ATPase-dependent acidification, though far less efficiently than that in wild-type macrophages^8^. This indicates that the *a*1- or *a*2 subunit isoform can partially rescue the loss of the *a*3 isoform. These observations suggest that various mechanisms at multiple levels are involved in regulation of the levels of the proton pump complex and its precise membrane distribution as well, for the maintenance of acid-ion homeostasis in various tissues.

Several studies have shown the underlying mechanism how cells sense the activities of proton pumps and acidification in the various subcellular compartments. Heteronuclear ribonucleoprotein-mediated stabilization of mRNA is involved in maintaining transcription level of *E*1 subunit^30^,^31^. Other studies have shown that sensing of organelle acidification regulates interaction of V-ATPase and cytoskeleton thus regulates subcellular distribution of V-ATPase^32^,^33^,^34^,^35^. Further, *G* subunit is a substrate for the proteasome-dependent proteolysis coupled with rab7/RILP endosome regulation^36^. These observations suggest that multiple regulatory mechanisms are involved in the determination of stable and transient levels of V-ATPase complex in response to the extent of acidification within various subcellular compartments as well as in the external milieu. Here, the current results suggested that the quantity of *G* subunits is regulated at the post-transcriptional level. Further studies into the mechanism responsible for quantity regulation of *G* as well as other subunits are needed to improve our understanding of the versatile physiological function of V-ATPase.

## Methods

### Reagents

Rabbit antibodies to *G*1, *G*2, *G*3, and anti-V-ATPase *a*1, *B*2 and *c* subunits were described previously^19^,^24^,^37^. Mouse antibody against β-catenin was obtained from Beckton Dickinson. Polymerase chain reactions (PCR) for bacterial artificial chromosome (BAC) modification and vector construction were performed with Phusion DNA polymerase (Finnzyme). Diagnostic PCR was performed using Ex Taq DNA polymerase (TaKaRa).

### Targeting construct

A targeting vector was created using recombinogenic strategy. A BAC clone (MGS1-205a4) containing the *Atp6v1g2* locus of mouse 129Sv strain was obtained from Genome Systems and introduced into *Escherichia coli* strain DY380, carrying an inducible homologous recombination system to obtain a strain 205a4/DY380^38^. A *lox* P-Zeo-*lox* P fragment with arms homologous to *Atp6v1g2* was amplified by PCR using the primer pair G2LZL-S1: 5′-ccaccccaactatcgggttactgtctagaaccatcgctcagggacacatcTCAACCATCATCGATCATAA-3′ and G2LZL-A1: 5′- tttgagttgggatatttctctgtggacggagctgacagaaggagtcactcAACCCTGAAGCTATCATAA-3′ (lowercase letters correspond to sequences in the 3′-untranslated region of the *Atp6v1g2*, and uppercase letters correspond to a template plasmid pMODloxZeo-ΔAmp3). The *lox* P-Zeo-*lox* P cassette vector^39^ was introduced into *E. coli* strain 205a4/DY380. Correct homologous recombination yielding a modified BAC containing *Atp6v1g2* locus with *lox* P-Zeo-*lox* P was screened via Zeocin resistance and confirmed by PCR analysis. Next, a linear pBSDT-AII fragment^39^ flanked by short *Atp6v1g2* segments was amplified using the primer pair G2DTAL: 5′-GCTCCTGGGTGGTAGCAGTGTGGTGCCTAGGGTTGCCGCTTACGgatcctagagcggccgccaccgcg-3′ and G2DTAR: 5′-CAGCACCTTGCCTCCTTGTGTAACAGCGGAGTGTCAATAAAATACTGGTGgatccggccggcccgggcga-3′ (lowercase and uppercase letters correspond to pBSDT-AII and *Atp6v1g2* sequences, respectively). The fragment was introduced into DY380 carrying the modified BAC to retrieve an approximately 7-kb fragment in the multicopy plasmid pBSDT-AII. The resultant plasmid was propagated in *E. coli* strain BH25.8 expressing *Cre* recombinase to remove the Zeo cassette and one of the *lox* P. Then, the vector was further modified with a *lox* P-*Frt*-*npt*-*lox* P-*Frt* cassette^39^ using a mini-transposon-mediated strategy to yield a targeting vector in which, *lox* P-*Frt*-*npt*-*lox* P-*Frt* was placed in intron 1–2 and the third *lox* P, in the 3′-UTR (Fig. 1). *Cre*-mediated recombination between the remotest *lox* P sequences produced an allele that was missing exon 2 and a part of exon 3, which encode the amino acid residues 28 to 118 of the *G*2 subunit.

### Immunoblot analysis

The brain and kidney were isolated and lysed in an extraction buffer containing 50 mM Tris-HCl (pH7.4), 1% sodium dodecyl sulphate (SDS) plus Complete proteinase inhibitors (Roche) and 1 mM phenylmethylsulfonyl fluoride. Protein concentration in the lysate was determined by the bicinchoninic acid colorimetric assay (Pierce). For the immunoblot analysis, 10 μg protein was loaded onto 5% to 20% SDS-polyacrylamide gel, electrophoresed, and immunoblotted as previously described^19^. Subunit *G* isoforms were detected by isoform-specific antibodies using the Pierce Western Blotting Substrate Plus system. Images were acquired and analyzed using the GE Healthcare LAS-4000 mini system^37^.

### Animals

All animal experiments were approved by the institutional committee (ISIR, Osaka University) and carried out in accordance with the rules and regulations of the institutions and the government. C57Bl/6 mice were purchased from SLC Japan. The targeting construct was introduced into mouse embryonic stem cells^40^ (R1, kindly provided by Dr. A. Nagy) by electroporation and recombinants carrying the modified allele in the *Atp6v1g2* locus were screened by PCR. The successful clones were injected into C57Bl/6 E3.5 blastocysts, to obtain chimeric mice, which were further crossed with C57Bl/6 mice. *Cre*-mediated recombination was performed in germ cells by crossing the chimeric mice with C57Bl/6 mice carrying an EIIa-promoter, *Cre*-recombinase transgene^41^.

### Quantitative real-time PCR (qPCR) for *G* subunit isoforms

We prepared brains and kidneys by macroscopic dissection from 1.5–2 month old mice (n = 3 (*Atp6v1g2*^+/−^ animals) or n = 4 (wild type and *Atp6v1g2*^*−*/*−*^ animals)). The tissues were quick frozen in liquefied nitrogen and kept under −80 °C until use, then total RNA was extracted using Qiagen RNeasy mini kit. cDNA was obtained from the 1500 ng of RNA (determined by spectrophotometry) by reverse-transcription in 20 μl reaction using a SuperScript VILO cDNA synthesis kit (Life Technologies). Primer efficiencies were determined from standard curves obtained by a dilution series of mixed pool of cDNAs derived from brain and kidney of wild-type animals (Table 2). The cDNA (corresponding to 1.5 ng–0.015 pg RNA) was subjected to qPCR in 25 μl of 1× SYBR Premix Ex Taq II (Tli RNase H plus) (TaKaRa), 200 nM primers, and amplification was monitored in Life Technologies/Applied Biosystems Prism7000. The plots were analyzed with ABI Prism software (ver. 1.1) to obtain quantification cycles (Cq). The linear regression plots (Cq against log(RNA amount)) gave r^2^ > 0.99 in the range of 1.5 ng–0.15 pg of RNA, and the primer efficiencies (E) were determined from the slope according to a formula E = 10^(−slope)^.

We quantified transcripts of reference genes *Gapdh*, *Actb*, *Tbp*, *Ppia* and *Ywhaz* in the cDNAs from brains and kidneys of wild-type, *Atp6v1g2*^+/−^, *Atp6v1g2*^*−*/*−*^ animals (Table 3). We then analyzed the amplification data by NormFinder algorithm^42^ in order to identify the best standard transcripts in the experiments (Table 4). Comparing expression levels in brain identified *Gapdh* as the most stable reference gene among the genotypes (stability value 0.066), following *Tbp* (0.133), *Actb* (0.134), *Ywhaz* (0.190), and *Ppia* (0.178). In kidney, *Actb* was the most stable (0.121), and *Gapdh* was the third (0.222). Although *Actb* appeared the best standard (0.193) if all the samples (both brain and kidney in three genotypes) were to be normalized, we chose *Gapdh* (0.233), the most stable standard reference in brain, as the normalizer in this experiment.

The relative amounts of *G* isoform transcripts were determined by the qPCR. The cDNA corresponding to 75 pg of RNA was subjected to qPCR in 25 μl reaction as specified above. The profile of thermal cycle was 1 min initial denature at 96 ˚C, 40 cycles of 96 ˚C 5 sec/60 ˚C 35 sec, and then melt curves were obtained gradually raising the temperature from 65 ˚C to 90 ˚C. The amplification plots were analyzed by the ABI Prism software to obtain Cq. In each samples, the relative amounts of *G* isoform transcript was calculated as ratio = E_*Gapdh*_^(Cq*Gapdh*)^/E_*Gn* isoform_^(Cq*Gn*)^. The changes in transcript levels among the genotypes were calculated as fold = E_Gn isoform, genotypes_^(Mean (Cq*Gn, wild)* − Mean (Cq*Gn, genotypes*))^/E_*Gapdh*, *genotypes*_^(Mean (Cq*Gapdh, wild*) − Mean (Cq*Gapdh*, *genotypes*))^^25^.

## Additional Information

**How to cite this article**: Kawamura, N. *et al.* Loss of *G*2 subunit of vacuolar-type proton transporting ATPase leads to *G*1 subunit upregulation in the brain. *Sci. Rep.* **5**, 14027; doi: 10.1038/srep14027 (2015).

## Figures and Tables

**Table 1:** Genotype of progeny from intercross of *Atp6v1g2*^+/−^ mice.

	**Genotype**		
	**+/+**	**+/−**	**−/−**
3–4 wks after birth	14	36	17
	(21%)	(54%)	(25%)

Genotype of progenies were determined by PCR of genomic DNA obtained from tail-tips.

**Table 2:** PCR primers used for quantification of transcripts.

**Gene**	**primer**	**sequence**	**efficiency (%)**	**amplicon size (bp)**	**intron length (bp)**
*Atp6v1g1*	G1-S201	5′-ccctcagcaatggcgagtcagtc-3′	105.4	137	3585
	G1-A201	5′-tcagcctgggcttcttctttgg-3′			
*Atp6v1g2*	VG2-S201	5′-aagcgggcagcggagaaggtg-3′	88.5	140	201
	VG2-A202	5′-gccgcctgctgcttgctct-3′			
*Atp6v1g3*	VG3-S201	5′-agaaaagaaaaggaaagcgactga-3′	96.5	315	4020
	VG3-A201	5′-tctatgcctatggacttggatgtg-3′			
*Gapdh*	GAPDH-S1	5′-tcccgtagacaaaatggtgaaggt-3′	106.2	184	1842
	GAPDH-A1	5′-tgtgccgttgaatttgccgtgagt-3′			

The oligonucleotides used for specific amplification of *G* subunit isoform cDNA are shown. The upstream and downstream primers are designed to anneal separate exons, thus minimizing the amplification of genomic DNA.

**Table 3:** PCR primers for the standard genes.

**gene**	**primer**	**sequence**	**efficiency (%)**	**references**
*Actb*	ActB-S1:	5′-gctgtattcccctccatcgtg-3′	106.4	43
	ActB-A1:	5′-cacggttggccttagggttcag-3′		
*Tbp*	Tbp-S1:	5′-ggcctctcagaagcatcacta-3′	119.4	43
	Tbp-A1:	5′-gccaagccctgagcataa-3′		
*Ppia*	Cyc-S1:	5′-agcactggagagaaaggatt-3′	103.2	44
	Cyc-A1:	5′-agccattcagtcttggcagt-3′		
*Ywhaz*	Ywhaz-S1:	5′-ttgagcagaagacggaaggt-3′	107.9	44
	Ywhaz-A1:	5′-gaagcattggggatcaagaa-3′		

The oligonucleotides used for amplification of candidate standard genes are described. Primer sequences were based on the references, modified to mouse sequence if necessary.

**Table 4:** Stability of the standard genes among genotypes.

**Gene**	**Stability**		
	**Brain**	**Kidney**	**Brain & Kidney**
*Gapdh*	0.066	0.222	0.233
*Actb*	0.134	0.121	0.193
*Tbp*	0.133	0.232	0.205
*Ppia*	0.178	0.305	0.283
*Ywhaz*	0.190	0.209	0.234

Stabilities of the candidate genes were examined. Cq was obtained in different samples, (brain and kidney, from the wild-type, *Atp6v1g2*^+/−^, and *Atp6v1g2*^−/−^ animals) and transformed to linear scale quantities by dCq method, then analyzed by NormFinder algorithm to calculate stability index^42^.
